# Comprehensive Evaluation of Biogas Slurry Fertility: A Study Based on the Effects of Biogas Slurry Irrigation on Soil Microorganisms and Enzyme Activities in Winter Wheat Fields

**DOI:** 10.3390/microorganisms13092054

**Published:** 2025-09-04

**Authors:** Dongxue Yin, Jiajun Qin, Baozhong Wang, Dongdong Chen, Zhiguang Dai, Xiaoli Niu, Jie Zhu, Fengshun Zhang

**Affiliations:** College of Agricultural Equipment Engineering, Henan University of Science and Technology, Luoyang 471000, China; qinj009@163.com (J.Q.); wbz666comeon@163.com (B.W.); chenddxuexi@163.com (D.C.); daizhiguang100@163.com (Z.D.); niuxiaoli88@126.com (X.N.); zj1270316684@163.com (J.Z.); 16627732157@163.com (F.Z.)

**Keywords:** biogas slurry, soil microorganisms, enzyme activities, comprehensive evaluation analysis, wheat

## Abstract

This study evaluates the impact of using biogas slurry (BS) instead of nitrogen fertilizer (NF) on wheat soil, and aims to provide an optimized fertilization strategy for green wheat production. Five fertilization modes were tested: basal fertilizer only (CK), NF at the full-bearing stage (CF), BS at the jointing stage + NF at the grouting period (S1), NF at the jointing stage + BS at the grouting period (S2), and BS at the full-bearing stage (S3). Wheat yield in S3 treatment was not significantly different from CF (9632.57 kg·ha^−1^), but significantly increased starch content by 23.39% (*p* < 0.05). Analysis of soil nutrient content showed that S3 treatment elevated ammonium nitrogen (AN) content by 98.30% during the harvest period and maintained the highest urease activity (686.45 μg·g^−1^·d^−1^). Microbial community analysis showed that the bacterial Shannon index under S3 treatment reached 7.09, and the abundance of Actinomycetes reached 39.40%. The fungal Simpson index was 0.02, lower than that of other treatments (*p* < 0.01). A comprehensive evaluation led to the conclusion that a complete replacement of BS with NF synergistically improves soil quick-acting nutrient levels, enhances soil enzyme activities, and sustains high microbial diversity, whilst maintaining wheat yield.

## 1. Introduction

Winter wheat represents one of China’s most significant grain crops, with total production reaching 137 million tons in 2023 [1]. Nitrogen fertilizer (NF) plays an essential role in winter wheat cultivation, directly affecting yield and quality. However, excessive NF use can lead to nitrate leaching into groundwater, contributing to water body eutrophication and compromising drinking water safety. Prolonged reliance on NF has also been shown to diminish microbial activity [2], resulting in soil acidification and degradation [3]. Moreover, the excessive application of NF has been associated with reduced crop yields. Wang et al. [4] found that the wheat yield of the treatment with a nitrogen application amount of 240 kg·ha^−1^ was 300 kg·ha^−1^ higher and the nitrogen uptake amount was 2.87% higher than that of the treatment with a nitrogen application amount of 276 kg·ha^−1^. Therefore, it is crucial to investigate sustainable and diversified alternatives to NF to enhance soil health and promote sustainable agriculture.

The ecological benefits of substituting NF with biogas slurry (BS) in agricultural production have been well documented. Research indicates that BS, as an organic fertilizer source, not only sustains crop yields, but also enhances the soil microecological environment and nutrient cycling efficiency through various mechanisms [5,6,7]. Tang et al. [8] reported that BS offers a balanced supply of nitrogen, phosphorus, potassium, and micronutrients throughout the wheat life cycle, improving wheat grain yield by 5–10% and facilitating the accumulation of soil organic matter (SOM) in long-term experiments. Yang et al. [9] demonstrated that the synergistic effects of amino acids and active enzymes in BS during the grain-filling process increased crude protein content in wheat grains to 14.24% and crude starch content to 78.40% when NF was replaced with BS.

In terms of soil ecological effects, Zhao et al. [10] found that replacing 50–75% of NF with BS significantly enhanced the microbial diversity index of inter-root soils (Shannon’s index increased by 0.38–0.62), with the abundance of ammonia-oxidizing and nitrogen-fixing bacteria increasing by 27% and 34%, respectively. This suggests that BS strengthens nitrogen transformation functions by regulating microbial community structure. Wang et al. [11] conducted a series of three-year positioning experiments, revealing that BS application significantly increased the proportion of water-stable macroaggregates (>0.25 mm) in the 0–20 cm soil layer from an initial 42.1% to 58.7%. Additionally, total soil porosity increased by 13.4%, attributed primarily to the colloidal stabilization of organic components (e.g., humic acid and dissolved organic carbon) in BS and their regulatory impacts on microbial activity. Culas et al. [12] found that BS fertilization can reduce greenhouse gas emissions (N_2_O reduction of 18–25%) and the risk of nitrogen leaching (30–50%) compared to traditional fertilizer models. These findings provide a theoretical foundation for developing a material cycle optimization model for the biogas–crop–soil system, offering a technical approach for managing agricultural surface pollution and efficiently utilizing nutrient resources.

Current research on substituting NF with BS primarily focuses on nutrient supply effects and crop yield evaluations. However, the synergistic response mechanisms of soil microbial and enzyme activities in winter wheat fields across different growth stages remain unclear, limiting the efficient utilization of BS. Therefore, based on previous research, a scientific hypothesis was proposed that the application of BS or NF during the different growth stages of winter wheat will have different effects on soil microbial community structure and enzyme activity. There are some fertilization strategies that can maintain soil sustainability while improving crop yield and quality. To verify this hypothesis, this study establishes a dynamic monitoring network based on the key fertility stages of winter wheat, namely, “jointing stage—flowering stage—grouting period—reaping period”. The dynamic evolution of soil microbial community structures and functional genes was analyzed using macro-genome sequencing technology under various alternative biomass treatments. And the response mechanisms of soil enzyme activities to different fertilization strategies were analyzed in depth. In addition, this study uses the combined analysis method of hierarchical analysis (AHP)–entropy weighting method (EWM) gray correlation analysis (GRA)–weighted gray correlation analysis (WGRA) to derive scientific and reasonable fertilization strategies from a more comprehensive and integrated perspective. Ultimately, the testable hypotheses proposed above were verified using the above methods. This paper provides scientific support for the efficient utilization of BS and the sustainable development of farmland.

## 2. Materials and Methods

### 2.1. Experimental Area Description

The experiment commenced on 12 April 2024 in Liuyang Village, Xin’an County, Luoyang City, a primary wheat-producing region in the Yellow and Huaihai Seas. The soil at the test site comprised clay loam with medium to high fertility, characterized by a soil moisture content of 7.33% (mass water content) in the 0–20 cm layer and a pH of 8.46. The area experiences a continental climate typical of temperate zones with an annual average temperature of 14.5 °C, a frost-free period of 223 days, and annual average precipitation and evapotranspiration of 578.2 mm and 1200.0 mm, respectively. The region benefits from ample sunshine and moderate rainfall, primarily concentrated between July and September. The soil nutrients are detailed in Table 1.

The wheat variety employed in the experiment was Luohan 20, and the BS was sourced from a conventional gas-producing biogas digester operated by the Lankeshan Cooperative in Xin’an County, Luoyang City, using a fermentation mixture of cow dung, pig manure, and crop stalks. BS was left to stand for 10 days before use, and was then filtered twice through a 100-mesh nylon net to prevent foreign matter in the BS from clogging the water pump or pipes. The nutrients of the biogas slurry are presented in Table 2.

### 2.2. Experimental Design

The fertilization regimen included basal fertilizer plus additional applications. The basal fertilizer comprised compound fertilizer, with the nitrogen application amount converted from methane liquid, in accordance with local farmers’ fertilization practices. A total of 750 kg·ha^−1^ of fertilizer was applied, with 450 kg·ha^−1^ designated as basal fertilizer and the remaining 300 kg·ha^−1^ evenly applied at the jointing stage (JS) and grouting period (GP). The experiment was structured with five treatments (Table 3): CK (as a control group, only base fertilizer was applied, and clean water was applied during the top dressing period.), CF (NF applied throughout the reproductive period), S1 (application of BS at the JS and NF at the GP), S2 (application of NF at the JS and BS at the GP), and S3 (application of biogas slurry throughout the reproductive period, excluding basal fertilizer). Each treatment was replicated three times in separate field plots, ensuring that the nutrient levels were consistent across all five fertilization regimens. Soil samples were collected at the JS, flowering stage (FS), GP, and reaping period (RP) of wheat.

### 2.3. Soil Sampling and Nutrition Analysis

Soil samples were collected during the JS, FS, GP, and RP growth stages of wheat, with five sampling points (0–20 cm) used for each treatment [13]. The collected soil was screened through a 2 mm sieve after removing impurities, and was thoroughly mixed. The soil was then divided into three parts: one part was stored at 4 °C, another part was frozen at −80 °C for soil DNA extraction, and the last part was dried in air for soil nutrient analysis [13].

Soil pH was determined using the potentiometric method, total nitrogen (TN) via the Kjeldahl method, soil organic matter (SOM) through potassium dichromate–ferrous sulfate titration, available phosphorus (AP) using the molybdenum antimony colorimetric method, available potassium (AK) via flame photometry, and ammonium nitrogen (AN) by potassium chloride leaching–flow analysis [14]. Wheat crude protein was assessed through the H_2_SO_4_-H_2_O_2_ digestion-AA3 flow analysis method [15], and amylose content was measured using the anthrone colorimetric method [16]. Wheat yield was estimated at a moisture content of 14% [17].

### 2.4. Determination of Soil Microorganisms and Enzyme Activity

Enzyme activities were assessed by pre-treating soil samples with kits from Solarbio, followed by measurement of catalase (CAT), urease (UE), sucrase (SC), and neutral phosphatase (NP) using fluorescence analysis in 96-well microtiter plates [18]. The results of enzyme activities were normalized. The soil samples were sent to Shanghai Meiji Biomedical Technology Co., Ltd., (Shanghai, China) for NovaSeq (Illumina, San Diego, CA, USA) macro gene sequencing using. The software fastp 0.20.0 was utilized to perform mass shearing of the adapter sequences at the 3′ and 5′ ends of the reads. Remove reads with a post-splicing length less than 50 bp, an average base quality score below 20, or containing N bases, and retain high-quality pair-end reads and single-end reads; select contigs ≥ 300 bp from the assembly results as the final assembly output. Using SOAPaligner 2.21, the high-quality reads of each sample were compared to a non-redundant gene set (95% identity), and gene abundance information was compiled for the corresponding samples.

### 2.5. Comprehensive Evaluation Method

A plethora of evaluation methods are in common usage. The hierarchical analysis method (AHP) is a systematic approach to decision-making that utilizes a structured framework to weigh and rank various criteria against a predetermined set of weights. This method is primarily applicable to scenarios requiring the comprehensive consideration of multiple decision-making factors, typically relying on experiential knowledge and expert insights to guide the decision-making process. With the objective of “developing an optimal fertilization strategy,” a three-tiered structure was established: Top level: Optimal fertilization strategy. Middle level: Core factors influencing the objective (e.g., “soil health, crop yield, and soil enzyme activity”). Bottom level: Specific quantifiable indicators under each core factor (e.g., “organic matter content,” “thousand-grain weight,” and “urease activity”). Subsequently, pairwise comparisons are conducted on the indicators in the middle layer to generate a judgment matrix, and, finally, the weights of each indicator are calculated [19,20]. Entropy Weighting Method (EWM) is a methodology that is based on the principle of information entropy. This is in contrast to AHP, which is based on subjective weighting. The EWM is able to objectively weigh indicators, and can be used to comprehensively assess multiple indicators. This can effectively reflect the importance of each indicator. Gray correlation analysis (GRA) is a methodological approach that is utilized to ascertain the extent of proximity between system factors. Weighted gray correlation analysis (WGRA) introduces the concept of weights on this basis, thereby reflecting the importance of each factor in the overall assessment [21].

In this study, the AHP was utilized for the determination of subjective weights, and the EWM and GRA methods were employed for the determination of objective weights. These weights were integrated into combined weights by a modified WGRA method for evaluating multiple metrics under each treatment. Consequently, the correlation between each treatment and the optimal solution was calculated. A higher correlation indicates that a certain irrigation strategy has a more pronounced combined effect on soil nutrients, enzyme activities, and microorganisms.

#### 2.5.1. Methods of Assigning Weights Using Analytic Hierarchy Process

Modeling the Hierarchy: A hierarchical structure typically comprises an objective layer, a factor layer, and an indicator layer, as stipulated by AHP. An evaluation system was developed to assess the sustainability of wheat growth and soil health. The development process involved the utilization of the AHP, incorporating the fundamental characteristics of the 20 indicators employed in the experiment. The resulting evaluation system is outlined in Table 4.

The Saaty 1–9 scale method (Table 5) was adopted to quantify the importance of each index. Establish a pairwise comparison matrix based on the importance of the different indicators.

(1) $$E={\left(e_{ij}\right)}_{m\times n}$$

*E* is a positive reciprocal matrix and $e_{ij}$ represents the importance of indicator $e_{i}$ relative to $e_{j}$ in the evaluation system, quantified as the judgment matrix value.

Geometric mean of each row of the judgment matrix:

(2) $$\bar{u_{j}}=\sqrt[{n}]{\prod_{i=1}^{n}e_{ij}},\;i=1,2,3,\ldots ,n$$

Normalization processing:

(3) $$u_{j}=\frac{\bar{u_{j}}}{\sum_{i=1}^{n}\bar{u_{j}}},\;i=1,2,3\ldots ,n$$

Calculation of the maximum eigenvalue of the matrix:

(4) $$\lambda_{max}\;=\;\sum_{i=1}^{n}\frac{{\left(Eu\right)}_{i}}{n_{i}}$$

Judgment matrix consistency check:

(5) $$CR=\frac{CI}{RI}$$

(6) $$CI=\frac{\lambda_{max}-1}{n-1}$$

In this equation, *CR* is the random consistency ratio; *CI* is the general consistency index; and *RI* is the average random consistency index, which can be found in a table according to the order of the matrix.

#### 2.5.2. Methods of Assigning Weights Using Entropy Weight Method

Standardization based on positive and negative attributes of selected indicators. For positive indicators,

(7) $${x^{\prime }}_{ij}={\;x}_{ij}-x_{min}/x_{max}-\;x_{min}$$

For negative indicators,

(8) $${x^{\prime }}_{ij}=x_{max}-{\;x}_{ij}/x_{max}-\;x_{min}$$

Calculate the weights. (1) Calculate the entropy value of the *j*-th indicator:

(9) $$e_{j}=-\frac{1}{ln(m)}\sum_{i=1}^{m}p_{ij}\;ln(p_{ij})$$

Calculate the weights of each indicator:

(10) $$W_{j}=\frac{1-e_{j}}{\sum_{i=1}^{m}\left(1-e_{j}\right)}$$

#### 2.5.3. Methods of Assigning Weights Using Gray Relational Analysis (GRA)

The gray relational coefficient for each treatment, with respect to the ideal solution, was calculated as follows:

Calculate the gray correlation:

(11) $$r_{j}=\frac{1}{m}\sum_{i=1}^{m}p_{ij}\frac{{∆}_{min}+\rho {∆}_{max}}{\left|x_{ij}-x_{0j}\right|+\rho {∆}_{max}}\;$$

Calculate the gray weights:

(12) $$w_{j}=\frac{r_{j}}{\sum_{i=1}^{n}r_{j}}$$

#### 2.5.4. Methods for Assigning Weights Using Portfolio Weights

Re-standardization of indicator data:

For positive indicators,

(13) $$r_{ij}={\;x}_{ij}-min(x_{j})/max(x_{j})-max(x_{j})$$

For negative indicators,

(14) $$r_{ij}=max(x_{j})-{\;x}_{ij}/max(x_{j})-min(x_{j})$$

Geometrically average the product of the three weights:

(15) $$U_{j}=\frac{\sqrt[{3}]{u_{j}\times W_{j}\times w_{j}}}{\sum_{j=1}^{n}\left(\sqrt[{3}]{u_{j}\times W_{j}\times w_{j}}\right)}$$

Resulting in the following final score:

(16) $$G_{j}=\sum_{j=1}^{n}\left(r_{ij}\times U_{j}\right)$$

### 2.6. Statistical Analysis

Microsoft Excel 2021 was employed to statistically organize the experimental data. The ‘ggplot2’ package in RStudio 3.5.3 was chosen for plotting, “vegan” package was utilized for α-diversity analysis of microbial communities, and SPSS 23.0 was used for one-way analysis of variance and significance testing.

## 3. Results

### 3.1. Wheat Yield Response to Different Fertilization Strategies

Table 6 illustrates the effect of various fertilization strategies on wheat yield and quality. No significant differences (*p* > 0.05) in effective spike number and thousand-grain weight were observed among treatment groups. The output of S3 was 9461.33 kg·ha^−1^, slightly lower than that of CF, which was 9632.57 kg·ha^−1^. The yields of both were significantly higher than that of CK (8970.67 kg·ha^−1^). Meanwhile, the standard deviation of S3 (±352.48) was significantly lower than that of CF (±720.53), indicating that the yield fluctuation of S3 was relatively small. In terms of amylose content, significant differences among treatments were noted (*p* < 0.001), with the highest content in BS, which was 23.39% greater than that in CF; the highest crude protein content was observed in S2, which was 12.56% higher than in S1, with significant differences among treatment groups (*p* < 0.001). Collectively, these results suggest that S3 can enhance both the yield and quality of wheat.

### 3.2. Response of Soil Nutrients to Different Fertilization Strategies

**Figure 1:**
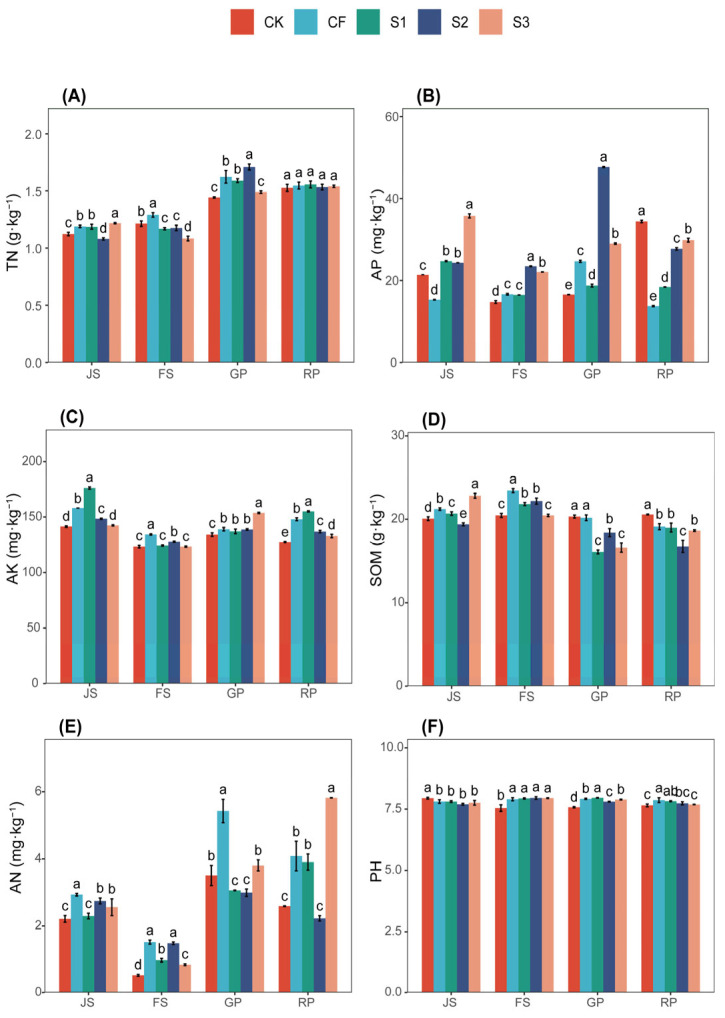
Soil nutrients treated differently. (**A**) TN, total nitrogen; (**B**) AP, available phosphorus; (**C**) AK, available potassium; (**D**) SOM, soil organic matter; (**E**) AN, ammonium nitrogen; (**F**) PH. JS, jointing stage; FS, flowering stage; GP, grouting period; RP, reaping period. The letters a, b, c, etc. in the picture represent the differences among various treatments within the same period. If the letters are the same, the differences are not significant; if the letters are different, the differences are significant. CK (water applied throughout the reproductive period), CF (NF applied throughout the reproductive period), S1 (application of BS at the JS and NF at the GP), S2 (application of NF at the JS and BS at the GP), and S3 (application of biogas slurry throughout the reproductive period, excluding basal fertilizer).

The effect of various fertilization treatments on soil TN content is depicted in Figure 1A. All treatment groups exhibited a general trend of increasing over time. Soil TN content was maintained at 1.08 to 1.71 g·kg^−1^ during wheat growth, with higher levels following the second fertilizer application compared to the first. The post-harvest TN content did not significantly differ among treatments, stabilizing in the range of 1.52–1.56 g·kg^−1^, with the highest content in S1, which was 1.96% greater than CK. Compared to pre-fertilization levels, the post-harvest TN content increased by 23.39%, 25.00%, 25.81%, 23.39%, and 24.19% in the order CK, CF, S1, S2, and S3.

Figure 1B illustrates the effect of different fertilization treatments on soil available phosphorus (AP). Most treatments during wheat growth exhibited lower AP than the initial value of 31.33 mg·kg^−1^. At the JS, S3 had the highest AP value of 35.75 mg·kg^−1^, an increase of 14.11% from the initial value, and 133.51% higher than the CF value at the same stage. At the GP stage, the AP value of S2 was 47.71 mg·kg^−1^, and, at the RP stage, the AP value of CK was 34.38 mg·kg^−1^. The AP values of these two treatments were both higher than the initial value, increasing by 52.28% and 9.74%, respectively. In the RP stage, CK had the highest AP value, which was 150.40%, 87.05%, 24.07%, and 15.25% higher than the CF, S1, S2, and S3, respectively, during the same period.

Figure 1C demonstrates the effect of different treatments on soil available potassium (AK) content. The AK levels of the treatments generally displayed a decreasing trend followed by an increase, with higher levels observed during the JS compared to other periods, and CK consistently having the lowest AK across all periods. The highest AK during wheat fertility was recorded in S1 during JS at 176.00 mg·kg^−1^, which was 23.66% higher than before fertilizer application and 24.53% higher than CK during the same period. The highest AK during RP was found in S1, whereas CK had the lowest at 155.04 and 127.28 mg·kg^−1^, respectively. S1 was 21.81% higher than CK, CF, S2, and S3, which were 4.83%, 13.20%, and 16.64% higher, respectively. Notably, AK levels decreased by −10.57%, 3.91%, 8.93%, −3.77%, and −6.61% across all treatments compared to pre-fertilization levels, indicating that S1 was effective in increasing AK content in soil.

Figure 1D presents the effect of different treatments on soil SOM content. CK maintained a stable SOM range of 20.07–20.57 g·kg^−1^ across all periods, which was lower than pre-fertilization levels. SOM peaked at 23.43 g·kg^−1^ during the FS period for CF, representing a 3.49% increase compared to pre-fertilization levels, and was 14.52% higher than values recorded for CK, S1, S2, and S3, which were 7.38%, 5.64%, and 14.52% higher, respectively. During the RP, the SOM levels for the treatments were 127.28 g·kg^−1^, 147.89 g·kg^−1^, 155.04 g·kg^−1^, 136.96 g·kg^−1^1, and 132.92 g·kg^−1^, which were −9.14%, −15.59%, −16.08%, −26.06%, and −17.58% lower than pre-fertilization levels, indicating that CK treatment resulted in less SOM depletion from the soil.

From Figure 1E, it can be observed that soil AN content across all treatments remained low during the FS period, significantly lower than the pre-fertilization levels of 2.93 mg·kg^−1^. The AN content of CF and S3 fluctuated considerably during the wheat RP, reaching their lowest values during the FS period at 1.52 mg·kg^−1^ and 0.85 mg·kg^−1^, which represented decreases of 48.30% and 71.09%, respectively, compared to pre-fertilization levels. The AN in CF peaked during the GP at 5.43 mg·kg^−1^, an increase of 84.69% compared to pre-fertilization levels, whereas S3 peaked during the RP at 5.83 mg·kg^−1^, representing a 98.30% increase from pre-fertilization levels. The AN levels during the RP were recorded at 2.59 mg·kg^−1^, 4.09 mg·kg^−1^, 3.91 mg·kg^−1^, 2.23 mg·kg^−1^, and 5.83 mg·kg^−1^, indicating that both S3 and CF facilitated the mineralization of TN in the soil into AN, which can be directly absorbed and utilized by wheat.

From Figure 1F, it is evident that soil pH consistently remained within the range of 7.54–7.97 during the wheat RP. The pH in CK showed a decreasing trend followed by a gradual recovery, whereas the other treatments exhibited an increasing and then decreasing trend. The pH in S1 peaked at 7.97 during the GP, an increase of 1.27% compared to levels prior to fertilizer application. During the RP, the pH values for the treatments were 7.65, 7.87, 7.83, 7.74, and 7.69, reflecting changes of −2.80%, 0.00%, −0.51%, −1.65%, and −2.29%, respectively, compared to pre-fertilization levels. This indicates the soil’s ability to self-regulate its pH.

### 3.3. Response of Soil Enzyme Activities to Different Fertilization Strategies

**Figure 2:**
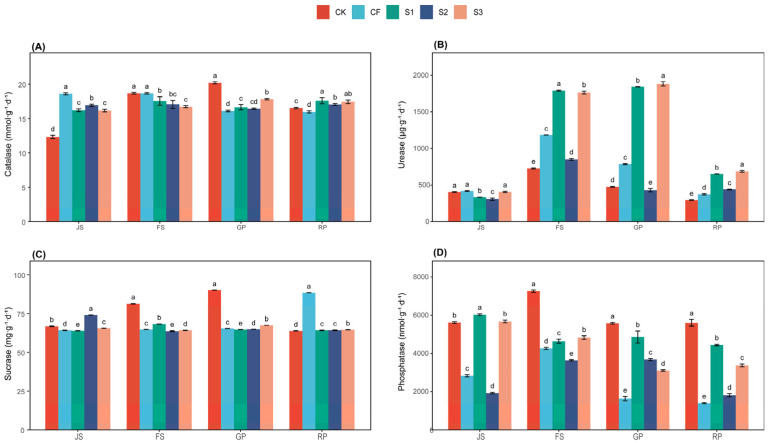
Soil enzyme activity under different treatments. (**A**) Catalase, CAT; (**B**) Urease, UE; (**C**) Sucrase, SC; (**D**) Neutral phosphatase, NP. The letters a, b, c, etc. in the picture represent the differences among various treatments within the same period. If the letters are the same, the differences are not significant; if the letters are different, the differences are significant. CK (water applied throughout the reproductive period), CF (NF applied throughout the reproductive period), S1 (application of BS at the JS and NF at the GP), S2 (application of NF at the JS and BS at the GP), and S3 (application of biogas slurry throughout the reproductive period, excluding basal fertilizer); JS, jointing stage; FS, flowering stage; GP, grouting period; RP, reaping period.

Catalase facilitates the decomposition of hydrogen peroxide, creating a conducive environment for root growth and development, thereby enhancing the plant’s capacity to absorb nutrients and water [22,23]. Significant differences were noted among the treatments during the wheat fertility period (Figure 2A). Soil CAT activity peaked at 20.19 mmol·g^−1^·d^−1^ during the GP in CK, which was 25.40%, 21.33%, 22.88%, and 13.30% higher than CF, S1, S2, and S3, respectively, during the same period. During the RP, S3 exhibited the highest CAT activity at 17.43 mmol·g^−1^·d^−1^, whereas the other treatments recorded 16.54 mmol·g^−1^·d^−1^, 15.96 mmol·g^−1^·d^−1^, 17.59 mmol·g^−1^·d^−1^, and 17.03 mmol·g^−1^·d^−1^. Thus, S3 effectively maintained higher levels of soil CAT activity.

UE catalyzes the hydrolysis of urea to yield ammonia and carbon dioxide, thereby supplying nitrogen that plants can directly absorb and utilize [24,25]. The effects of various fertilization treatments on soil UE activity are illustrated in Figure 2B. During the wheat fertility period, UE activity across all treatments exhibited a trend of initially increasing followed by decreasing. Notably, the activity levels were higher during the FS and GP phases, with significant differences among treatments (*p* < 0.001). The UE activity in S3 consistently exceeded that of other treatments throughout different periods, peaking at 1881.90 μg·g^−1^·d^−1^ during GP. The UE activities during RP for the treatments were recorded as 284.25 μg·g^−1^·d^−1^, 373.63 μg·g^−1^·d^−1^, 649.95 μg·g^−1^·d^−1^, 438.85 μg·g^−1^·d^−1^, and 686.45 μg·g^−1^·d^−1^. The UE of S1 was consistently slightly lower than that of S3, indicating that biomass application during the JS period could sustain elevated soil urease activity.

SC facilitates the hydrolysis of sucrose in the soil, resulting in glucose and fructose production, which increases the content of soluble sugars in the soil and provides a carbon and energy source that soil microorganisms and plants can readily absorb and utilize [26,27]. Significant differences were noted among the treatments during the wheat fertility period. S1 and S3 exhibited fluctuating SC activity around 65.00 mg·g^−1^·d^−1^ throughout the fertility period, whereas soil SC activity reached a peak of 90.09 mg·g^−1^·d^−1^ in CK during GP. The observed SC activities among the treatments during RP were 63.84 mg·g^−1^·d^−1^, 88.46 mg·g^−1^·d^−1^, 64.17 mg·g^−1^·d^−1^, 64.20 mg·g^−1^·d^−1^, and 64.49 mg·g^−1^·d^−1^, respectively.

NP enhances the conversion and utilization of phosphorus fertilizers applied to the soil and mitigates phosphorus losses [28,29]. Throughout the wheat fertility period, CK, CF, and S2 displayed an increasing and then decreasing trend, whereas S2 and S3 exhibited a decreasing trend, with significant (*p* < 0.001) among treatments. Soil NP activity peaked at CK during FS, measuring 7258.00 nmol·g^−1^·d^−1^. At RP, CK demonstrated the highest NP activity at 5597.54 nmol·g^−1^·d^−1^, which was 301.44%, 26.26%, 209.65%, and 66.02% higher than the other treatments. This finding correlates with the observation that the pH of CK consistently remained around 7.5.

### 3.4. Response of Soil Microorganisms to Different Fertilization Strategies

**Figure 3:**
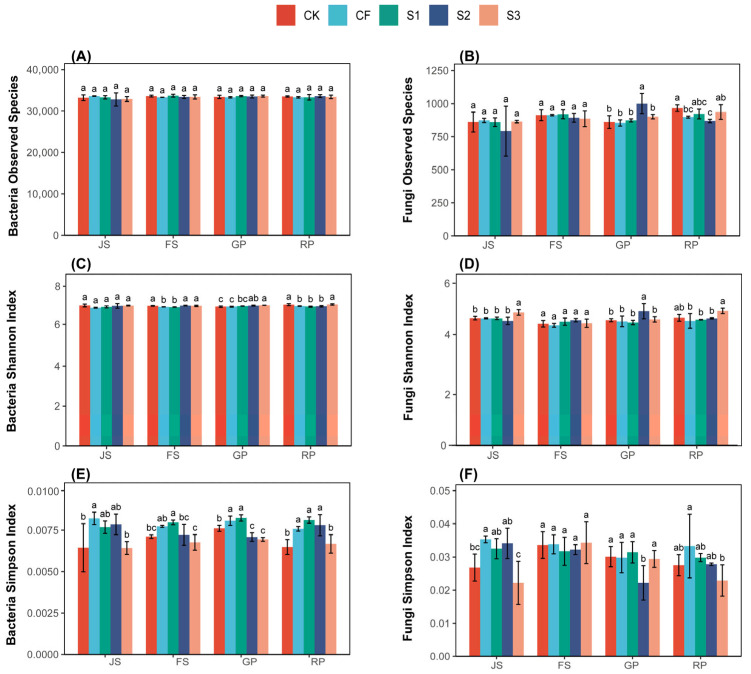
Soil microbial diversity under different treatments. Bacteria observed species (**A**), shannon index (**B**) and simpson index (**C**). Fungi observed species (**D**), shannon index (**E**) and simpson index (**F**). The letters a, b, c. in the picture represent the differences among various treatments within the same period. If the letters are the same, the differences are not significant; if the letters are different, the differences are significant. CK (water applied throughout the reproductive period), CF (NF applied throughout the reproductive period), S1 (application of BS at the JS and NF at the GP), S2 (application of NF at the JS and BS at the GP), and S3 (application of biogas slurry throughout the reproductive period, excluding basal fertilizer). JS, jointing stage; FS, flowering stage; GP, grouting period; RP, reaping period.

Panels A, B, and C in Figure 3 illustrate the changes in bacterial communities in wheat field soil during the wheat fertility period, whereas panels D, E, and F depict changes in fungal communities. For bacteria, the different fertilization treatments did not induce significant changes in the observed species in wheat field soil. The Shannon index across all treatments fluctuated around 7 throughout the RP, with S3 maintaining a consistently higher level; the highest index for S3 was 1.58% greater than the lowest for S1 during RP. The Simpson index for S1 consistently exceeded that of other treatments across different periods, and, during RP, S1 was 25.15% higher than the lowest CK. For fungi, no significant differences in observed species were found among the treatments. During RP, S3 exhibited the highest Shannon index, which was 8.02% greater than the lowest CF. S3 registered the lowest Simpson index, which was 45.41% lower than the highest CF. Overall, S3 exhibited the highest fungal diversity.

**Figure 4:**
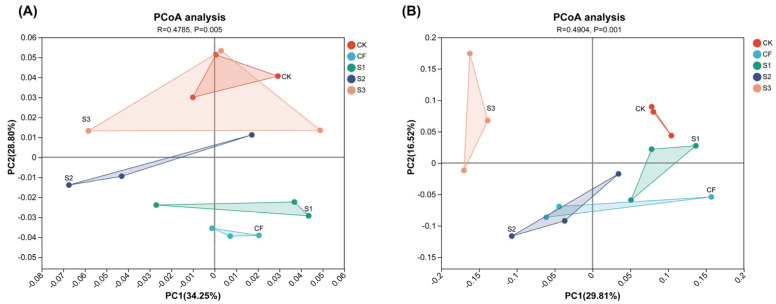
Principal component analysis of soil bacterial community (**A**) and fungal community (**B**) under different fertilization strategies.

A principal component analysis of microbial community abundance was conducted, with the results presented in Figure 4. The variance contributions of the first and second principal components regarding the bacterial community were 34.25% and 28.80%, respectively, yielding a combined contribution of 63.05%, which effectively elucidates the correlation and variability among treatments, facilitating differentiation between them. Significant differences were observed among treatments. The variance contributions of the first and second principal components regarding the fungal community were 29.81% and 16.52%, respectively, with a combined contribution of 46.33%, adequately explaining the correlation and variability among different treatments. The differences among treatments were highly significant.

**Figure 5:**
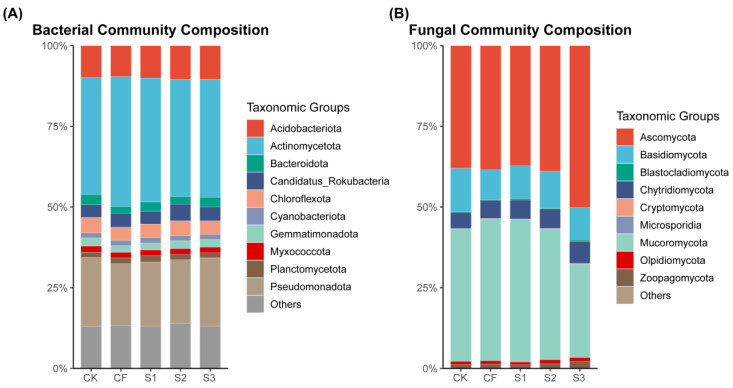
Soil bacterial community phylum abundance (**A**) and fungal community phylum abundance (**B**) under different fertilization strategies.

The dominant bacterial phyla under different fertilization treatments included *Actinomycetota* (36.28–40.26%), *Pseudomonadota* (19.19–21.39%), and *Acidobacteriota* (9.59–10.45%) (Figure 5A). Compared to CK, CF significantly enhanced the abundance of *Actinomycetota*, whereas S2 notably increased the abundance of *Acidobacteriota*. The dominant fungal phyla across different fertilization treatments consisted of *Ascomycota* (37.22–50.19%), *Basidiomycota* (9.16–13.40%), and *Mucoromycota* (28.85–44.08%) (Figure 5B). The highest abundance of *Ascomycota* was observed in S3. *Mucoromycota* abundance was the lowest, with other phylum-level fungal abundances in S3 consistently exceeding those of other treatments. *Ascomycota* abundance showed no significant differences among CF, S1, and S2, whereas *Basidiomycota* abundance was significantly lower than that of CK. *Mucoromycota* abundance in CF and S1 was significantly higher compared to other treatments.

### 3.5. Relationship Between Soil Microorganisms and Environmental Factors

**Figure 6:**
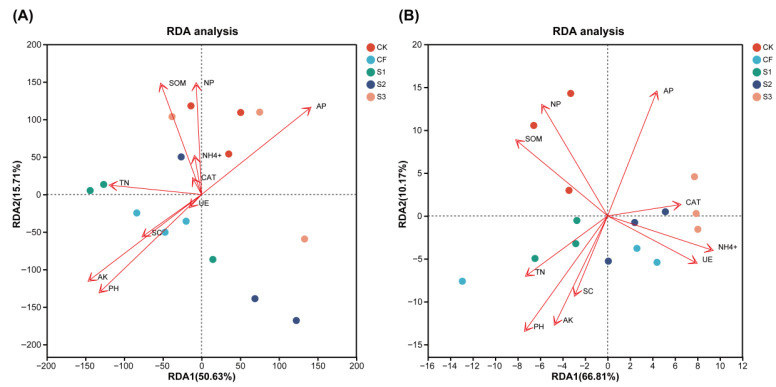
Redundancy analysis (RDA) ordination diagrams describing the relationship between bacterial (**A**) and fungal (**B**) communities and wheat soil properties. TN, total nitrogen; AP, fast-acting phosphorus; AK, fast-acting potassium; NH4+, ammonium nitrogen; SOM, soil organic matter; CAT, catalase activity; UE, urease activity; NP, neutral phosphatase activity; SC, sucrase activity.

Redundancy analysis (RDA) was performed to elucidate the association between dominant bacterial and fungal community composition and soil factors (TN, AP, AK, NH_4_^+^, SOM, CAT, UE, NP, SC, pH) (Figure 6). The two main axes accounted for 66.34% and 76.98% of the total variance in bacterial and fungal community differences, respectively. AP and NP exerted a pronounced influence on bacterial and fungal community structure. The bacterial sample points from CF were distributed around TN, AK, PH, and SC, indicating that the microbial structure of CF is highly correlated with these environmental factors. In terms of the fungal community, the sample points for S3 were primarily concentrated in the upper right, which markedly differed from the distribution area of the sample points from other treatments, indicating substantial differences between S3 and the other treatments.

**Figure 7:**
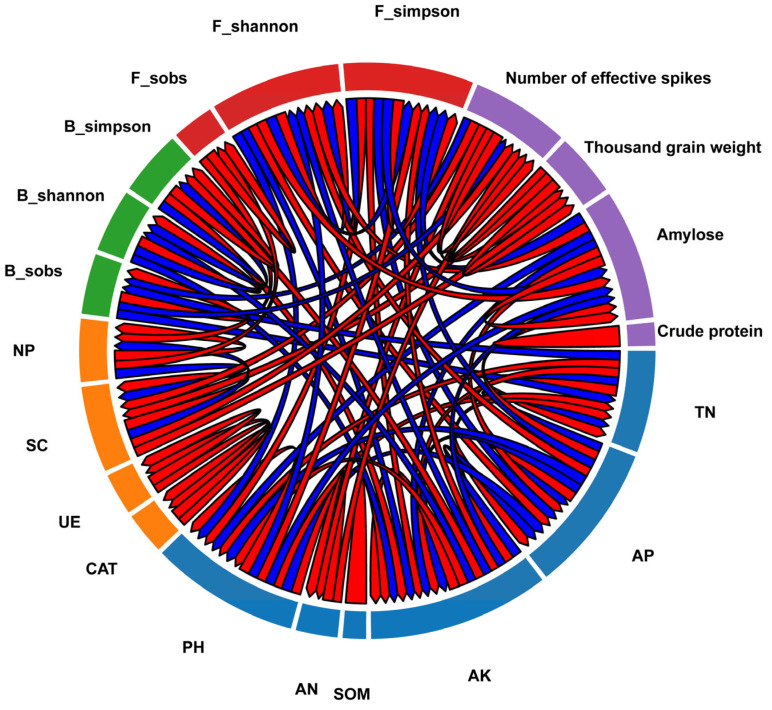
Correlations between soil nutrients, microorganisms, and wheat yield (red strings indicate positive correlations, blue strings indicate negative correlations, and the width of the strings reflects the strength of the correlation).

As demonstrated in Figure 7, a positive correlation between TN and thousand-grain weight was observed, indicating that elevated soil nitrogen levels contribute to wheat grain filling. Furthermore, a positive correlation between AK and B_shannon was identified, suggesting that increased potassium content in soil may promote the diversity of bacterial communities. Additionally, a reciprocal relationship between F_shannon and PH was observed. Finally, the number of effective spikes of wheat was found to be positively correlated with the Shannon diversity of the fungal community (F_shannon), indicating that a rich fungal community may be conducive to wheat spike development. These associations provide a valuable perspective on the intricacies of soil ecosystems and the optimization of wheat cropping management.

**Figure 8:**
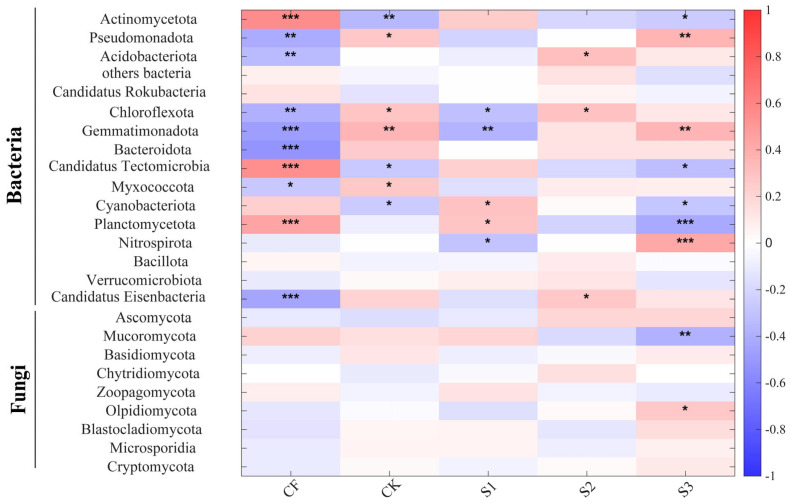
Spearman’s correlation between relative abundance of soil bacterial and fungal phyla and fertilization strategies (* for *p* < 0.05, ** for *p* < 0.01, *** for *p* < 0.001).

As illustrated in Figure 8, a significant Spearman correlation exists between diverse fertilization strategies and bacterial and fungal communities. It was observed that not all fertilization strategies exhibited a strong correlation with fungi; two treatments, CF and S3, demonstrated stronger effects on microorganisms compared to the others. It is noteworthy that CK and S3 exhibited diametrically opposed effects on the same microorganisms. The present study demonstrates that the CK treatment significantly promoted the growth of *Pseudomonadota* and *Acidobacteriota*. Furthermore, the CF treatment was found to be significantly positively correlated with *Actinomycetota* and *Candidatus_Tectomicrobia*. In addition, the S1 treatment was found to be significantly positively correlated with *Gemmatimonadota* and *Bacteroidota*. The second-season (S2) treatment demonstrated a significant positive correlation with *Planctomycetota* and *Nitrospirota*, while the third-season (S3) treatment exhibited a significant promotion in the growth of *Candidatus_Eisenbacteria* and *Mucoromycota*, along with a substantial inhibition in the growth of *Cryptomycota*.

**Figure 9:**
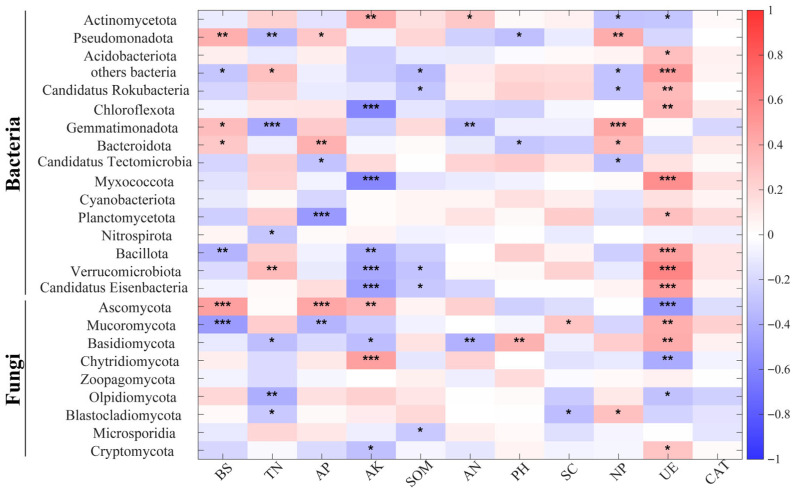
Spearman’s correlation between relative abundance of soil bacterial and fungal phyla and environmental factors (* for *p* < 0.05, ** for *p* < 0.01, *** for *p* < 0.001).

As illustrated in Figure 9, a Spearman correlation heat map is presented which demonstrates the relationship between soil nutrients and soil microbial communities. As illustrated in Figure 9, a significant positive correlation was observed between BS and *Pseudomonadota* and *Ascomycota*, while a significant negative correlation was identified between BS and *Mucoromycota*, which exerted a substantial influence on the microbial community structure. The effects of AK and UE on the same microorganisms were consistently opposite, with AK exhibiting a negative correlation with the dominant microorganisms and UE demonstrating a positive correlation. In the bacterial community, the dominant species *Actinomycetota* demonstrated a positive correlation with TN, AK, AN, and SOM and a negative correlation with BS, AP, NP, and UE. In contrast, *Pseudomonadota* exhibited a positive correlation with BS, AP, SOM and NP and a negative correlation with TN, AN, PH, and UE. Among the fungal communities, *Ascomycota* was positively correlated with BS, AP, AK, and AN and negatively correlated with PH and UE. *Mucoromycota* was positively correlated with TN, SC, and UE and negatively correlated with BS, AP, AK. It can be concluded that BS, TN, AP, AK, SOM, NP, and UE are the important influences on microbial community structure.

**Figure 10:**
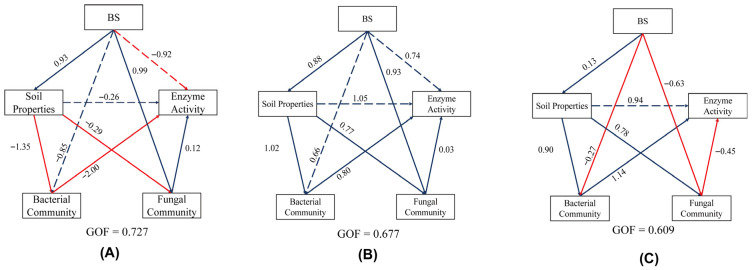
Partial least squares path model (PLS—SEM) analysis of the soil properties and soil microorganisms affected by biogas slurry under different fertilization strategies (the blue arrow represents a significant positive impact, the red arrow represents a significant negative impact, and the implementation represents a direct impact relationship; the dashed line represents an indirect impact relationship (**A**) as S1, (**B**) as S2, and (**C**) as S3).

A PLS—SEM analysis was further used to investigate the association between soil nutrients and microbial community characteristics under varying fertilization patterns (Figure 10). The results reveal that biogas in S1 negatively affected the bacterial community (−0.85) and enzyme activity (−0.92), whereas soil nutrients adversely affected microbial community structure (−1.35) and enzyme activity (−0.26). This may be because the application of NF during the GP increased soil nutrients, which were not effectively utilized by the bacterial community. This led to an imbalance in the microbial community and inhibited enzyme activity [30]. In contrast, all effects were significantly positive in the S2 treatment, where soil nutrients strongly influenced bacterial community structure (1.02) and enzyme activity (1.05). In S3, biosolids had negligible effects on enzyme activity and negatively affected both bacterial community (−0.27) and fungal community (−0.63), whereas bacterial community (0.90) and fungal community (−0.45) were strongly affected by soil nutrients. This may be because BS slow-release nutrients can basically meet nutrient requirements, but the microorganisms in BS have disrupted the structure of the soil microbial community [31].

### 3.6. Comprehensive Evaluation Analysis

#### 3.6.1. Determination of Single Indicator Weights Using the Analytic Hierarchy Process

**Figure 11:**
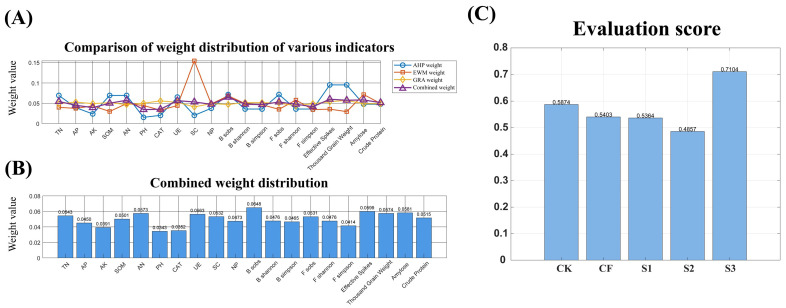
Comparison of weight distribution of various indicators (**A**), Combined weight distribution (**B**), Evaluation score (**C**) using the AHP-EWM-GRA-WGRA evaluation method.

The judgment matrices of target level A, factor level B, factor level B1–indicator level C, factor level B2–indicator level C, factor level B3–indicator level C, and factor level B4–indicator level C are obtained by reference to Table 4 and Equations (1)–(5). The local and overall weights are then calculated. The local weights are displayed in Table 7, Table 8, Table 9, Table 10, Table 11 and Table 12 and the overall weights are displayed in Figure 11. When the consistency coefficient CR is less than 0.1, it indicates that the consistency of the judgment matrix is high and the results of the hierarchical total ranking are satisfactory. The entropy weight method is utilized to ascertain the weights of individual indicators.

#### 3.6.2. Determination of Single Indicator Weights Using the Entropy Weight

Regarding the method based on Formulas (6)~(10), the weights of the different evaluation indicators can be determined using the entropy weight method, as shown in Figure 11.

#### 3.6.3. Determination of Single Indicator Weights Using the Gray Relational Analysis

Method Based on Formulas (11) and (12), the weights of the different evaluation indicators can be determined by using gray relational analysis, as shown in Figure 11.

#### 3.6.4. Combined Weight Assignment

The process of weight assignment was undertaken with the implementation of multiplicative synthetic normalization. The results of combining the assignments by the multiplicative synthetic normalization method (Formula (15)) are demonstrated in Figure 11. The relative importance of the indicators for assessing the effect of coordinated control under different treatments was determined by assigning a ranking to each indicator. The ranking was as follows: Effective Spikes > Thousand-Grain Weight > Amylose > Crude Protein > TN > AP > AK > SOM > AN > PH > CAT > UE > SC > NP > B_sobs > B_shannon > B_simpson > F_sobs > F_shannon > F_simpson. As illustrated in Figure 10 and Figure 11, which depict the final combination weights and final scores for each treatment, respectively, it can be concluded that the fertilizer strategy of irrigating the muck during the entire reproductive period was significantly superior to the other fertilizer strategies.

## 4. Discussion

### 4.1. Effect of Replacement of NF by BS on Quality and Yield of Wheat

As an organic fertilizer, BS not only enhances soil fertility, but also reduces reliance on NF, thereby mitigating the risk of environmental pollution [32,33]. The impacts of various fertilization strategies on wheat yield and quality were significant, with both CF and S3 markedly increasing the number of effective spikes and thousand-grain weight. The CF treatment yielded a high effective number of spikes and thousand-grain weight, but the lowest straight-chain starch content, which is directly attributable to the “carbon-nitrogen competition effect” of excess nitrogen inhibiting starch synthase activity [34,35]. The S2 treatment maintained a high effective spike number and crude protein content by fulfilling the peak nitrogen demand during tillering through fertilizer application at the JS and facilitating slow release of nitrogen and phosphorus from the marsh solution during GP [36]. S3 mitigated the peak activity of the nitrogen-metabolizing enzyme GS through the gradual release of nitrogen, delaying the inhibition of the carbon metabolism by the nitrogen metabolism (carbon and nitrogen are in competition: in a high-nitrogen environment, carbon flows to the nitrogen metabolism; in a low-nitrogen environment, carbon shifts to starch), thereby enhancing straight-chain starch content, albeit resulting in lower crude protein levels [36,37]. This aligns with Zhang et al. [38], who showed that inhibiting GS activity blocks nitrogen assimilation and thus attenuates—or even eliminates—the carbon–nitrogen competition effect. Despite S3 exhibiting the highest content of straight-chain starch, the lower crude protein content and effective number of spikes compared to CF and S2 reflect the delayed nitrogen release from methane relative to the rapid growth demands of wheat during the pre-reproductive stage. These findings align with results from other related studies [39,40]. The research indicates that the spatial and temporal distribution of the carbon and nitrogen metabolisms can be effectively regulated through rational organic–inorganic fertilization, providing a theoretical foundation for sustainable agriculture.

### 4.2. Effect of Replacement of NF by BS on Soil Nutrients at Different Fertility Periods

Soil fertility is a fundamental indicator of plant productivity, and scientific fertilization represents a critical agronomic strategy to maintain and enhance soil fertility [41]. This study demonstrates that soil nutrient content decreased across all treatments compared to the pre-fertilization period. However, TN content exhibited a continuous increase throughout the reproductive period and did not significantly differ between treatments (*p* > 0.05). This phenomenon was primarily attributed to the slow-release nitrogen source of the basal fertilizer and the inherent nitrogen pool from soil mineralization, which together mitigated nitrogen loss [42,43]. The AN content of CF was consistently maintained at a high level, whereas that of S3 did not peak until the RP, indicating that the slow release of nitrogen from the digestate was both gradual and prolonged, sufficiently meeting the nutrient demands of wheat during the middle and late stages of reproduction. Notably, the AN content of S2 was lower than that of the other treatments during the RP, suggesting that the application of digestate during the filling phase was inadequate to meet the nitrogen requirements of wheat at that time, resulting in a lower yield for S2 compared to the other treatments. In terms of AP, the S3 treatment maintained a relatively stable level, closely associated with the dual mechanisms of active phosphatase in the digestate which promote the mineralization of organic phosphorus and humic acid, thereby inhibiting phosphorus fixation [44,45]. The AK exhibited a trend of initial decrease followed by an increase across all treatments, with reductions observed during the JS, indicating that the rapid growth of wheat stems and leaves during the nodulation period resulted an increased demand for potassium. These findings align with the study conducted by Li et al. [46]. Furthermore, all treatments showed an increasing trend in soil pH at the JS, with a subsequent decline during the RP, although the pH consistently remained within a slightly alkaline range of 7.0–8.0. This pattern can be attributed to the dynamic equilibrium between the short-term acidifying effects of NF and the continuous release of alkaline substances from organic fertilizers [47,48]. SOM can characterize the stability and self-regulating ability of soil microbial communities in maintaining function in the face of external disturbances [49]. CF exhibited an increasing and then decreasing trend, reflecting the accelerated microbial mineralization of pre-existing organic matter due to the stimulating effects of fertilizers, leading to net depletion [50,51]. The decrease in SOM observed in S1 during the GP indicated that the microbial–substrate equilibrium was disrupted by fertilizer application, whereas the rebound of S1 during the RP signified the recovery of systemic self-regulation functions. Interestingly, S2 demonstrated a continuous depletion of SOM from the GP, potentially associated with the reorganization of the microbial community and enhanced metabolic activity triggered by the digestate input. In contrast, SOM content in S3 reached its lowest value during the GP, but rebounded during the RP, indicating that microbial community self-regulation was completed and that related functions were restored [49].

### 4.3. Effect of Replacement of NF by BS on Soil-Related Enzyme Activities at Different Fertility Periods

Soil enzymes, primarily derived from plant, animal, and microbial cells, play a crucial role in the soil nutrient cycling process [2,52]. RDA and partial least squares structural equation modeling (PLS-SEM) revealed interactions among soil nutrients, enzyme activities, and bacterial and fungal communities following various treatments. The UE and NP activities in S3 remained elevated throughout different fertility periods, likely due to the rich organic matter and slow-release nutrients in S3, which provided a continuous carbon and nitrogen source for soil microorganisms, thereby enhancing related enzyme activities. The CF treatments significantly elevated UE and SC activities in the short term, particularly during the fertilizer application period. This increase may be attributed to the rapid release of fast-acting nutrients in NF, which provided abundant nitrogen and carbon sources for microorganisms, stimulating UE and SC production. The staged application of BS influenced the activities of specific enzymes by regulating the timing of fertilization; for instance, S1 preferentially activated UE, enhancing sustained nitrogen mineralization, whereas S2 elevated CAT and NP activities, indicating that BS activated the function of phosphate-solubilizing bacteria, whereas NF inhibited enzyme activities due to negative feedback from phosphorus availability [53]. In summary, BS optimizes soil enzyme activity through the dual mechanisms of “carbon source driving” and “functional bacteria screening,” whereas NF tend to create a “nutrient pulse” effect. The disparity between the two highlights the potential of organic management to regulate soil enzyme activity over the long term, with significant implications for soil fertility and crop growth.

### 4.4. Effects of Replacing NF with BS on the Structure of Soil Bacterial Communities at Different Fertility Periods

Microorganisms are integral to soil and crop nutrient cycling [54,55], and the microbial community is sensitive to soil structure and nutrient status [56]. Principal coordinate analysis (PCoA) confirms this association. The diversity and abundance of microbial communities can be assessed using the α-diversity index [57]. In terms of α-diversity, the bacterial Shannon indices for S3 reached 7.05 ± 0.00 and 7.09 ± 0.03 during the GP and RP, respectively, significantly exceeding the indices of 6.98 ± 0.02 and 7.01 ± 0.01 for CF (*p* < 0.05). The Simpson index remained stable at 0.01, suggesting that BS fosters ecological niche differentiation for various functional bacterial communities through continuous organic carbon inputs, thereby reducing competitive exclusion [33,45]. Distinct fertilization treatments notably influenced the structure and functional dynamics of soil microbial communities. The results indicate that *Actinomycetota*, *Pseudomonadota*, and *Acidobacteriota* constitute the dominant phyla in the bacterial community (comprising 60–80% collectively), whereas *Mucoromycota* and *Ascomycota* dominate the fungal community (accounting for 75–90% collectively). CF enhanced the soil microbial community by increasing the abundance of *Actinomycetota* (40.7%) and *Ascomycota* (38.5%), thereby enhancing soil nitrification and organic matter mineralization capacity. However, the high nitrogen input accelerated the nitrification process, disrupting the metabolism of *Pseudomonadota* (19.19%), a key participant in the nitrogen cycle. S1 preferentially activated *Mucoromycota* (44.08%), leveraging its capacity to rapidly assimilate simple carbon sources to promote crop growth [58]. Conversely, S2 treatment synergistically facilitated nitrogen conversion and carbon turnover by activating the metabolic activities of *Pseudomonadota* (19.7%) and *Acidobacteriota* (10.5%), whereas preserving the symbiotic functions of *Mucoromycota* (40.4%) and *Basidiomycota* (11.4%), thereby forming a more stable microbial interaction network [59]. *Ascomycota* (45.3–58.3%) was predominant in S3, inhibiting *Trichoderma* spore germination through spatial occupancy and antibiotic secretion [60]. These findings indicate that various fertilization treatments exert differential effects on the functioning of soil microbial communities, providing a theoretical basis at the microbiome level for sustainable agricultural development.

### 4.5. Comprehensive Evaluation Using AHP-EWM-GRA-WGRA

AHP-EWM-GRA-WGRA is a pivotal instrument for optimizing fertilization strategies and balancing wheat yield quality and sustainable soil use. However, previous studies have predominantly relied on field trials to analyze the indicator patterns, and the assessment methods are often subjective or objective single-assignments, which have certain biases and are prone to leading to excessive attention being paid to certain indicators while ignoring other important factors [61]. In this study, an AHP-EWM-GRA-WGRA comprehensive assessment model was constructed. The hierarchical analysis method (AHP) was used to quantify expert experience, establish a hierarchical structure of indicators, and carry out subjective assignments. The entropy weight method (EWM) was used to carry out objective assignments. Weights were assigned to 20 indicators, covering soil nutrients, enzyme activity, microbial diversity, and wheat quality and yield. The gray correlation method (GRA) was used to calculate the relationship between indicators and ideal solutions. The following calculation was performed in order to determine the dynamic association strength between the indicators and the ideal solution. The construction of the association weights was achieved by means of multiplicative synthetic normalization. These weights were then applied to weighted gray correlation analysis (WGRA). The model provides a quantitative tool for evaluating the fertilization strategy of replacing NF with BS, and its combination of subjective and objective weights maximally eliminates the assessment bias caused by subjective preference and data fluctuation. This can provide methodological support for the green and sustainable development of agriculture.

## 5. Conclusions

The experimental results indicate that the wheat yield from the S3 treatments was marginally lower than from the CF treatment. However, the quality of the wheat was significantly enhanced. In relation to soil nutrients, the substitution of NF with BS has been demonstrated to engender a substantial augmentation in the levels of AP and AN. The bacterial dominant phyla were *Actinomycetota*, *Pseudomonadota*, and *Acidobacteriota*, and the fungal dominant phyla were *Ascomycota*, *Basidiomycota*, and *Mucoromycota*, in all treatments. The S3 treatments demonstrated the capacity to maintain high diversity. The activities of UE, CAT, SC, and NP were found to be consistently higher in the S3 treatment under the influence of the biogas slurry. A thorough AHP-EWM-GRA-WGRA evaluation determined that the S3 treatment exhibited notable efficacy in enhancing wheat quality, improving soil nutrients, preserving microbial diversity, and augmenting soil enzyme activities. These outcomes suggest that the S3 treatment can effectively promote wheat growth and soil health, thus offering a viable alternative to nitrogen fertilizers.

## Figures and Tables

**Table 1:** Main properties of soil.

pH	TN (g·kg^−1^)	AP (mg·kg^−1^)	AK (mg·kg^−1^)	SOM (g·kg^−1^)	AN (mg·kg^−1^)
7.87 ± 0.032	1.24 ± 0.0055	31.33 ± 0.089	142.33 ± 0.58	22.64 ± 0.29	2.94 ± 0.088

TN, total nitrogen; AP, available phosphorus; AK, available potassium; SOM, soil organic matter; AN, ammonium nitrogen.

**Table 2:** Main properties of biogas slurry.

pH	Total Solids Concentration	Volatile Solids	AP (mg·L^−1^)	AK (g·L^−1^)	TN (mg·L^−1^)	SOM (g·L^−1^)
8.35	0.43%	34.92%	18.08	0.84	375.54	1.11

AP, available phosphorus; AK, available potassium; TN, total nitrogen; SOM, soil organic matter.

**Table 3:** The amount of biogas digestate and fertilizer used in different treatments.

Treatment	JS			GP		
	Water L·ha^−1^	Amount of Nitrogen Fertilizer kg·ha^−1^	Amount of Biogas Digestate L·ha^−1^	Water L·ha^−1^	Amount of Nitrogen Fertilizer kg·ha^−1^	Amount of Biogas Digestate L·ha^−1^
CK	33,960	0	0	33,960	0	0
CF	33,960	150	0	33,960	150	0
S1	16,980	0	16,980	33,960	150	0
S2	33,960	150	0	16,980	0	2264
S3	16,980	0	16,980	16,980	0	16,980

JS, jointing stage; GP, grouting period.

**Table 4:** Hierarchical analysis method evaluation system.

Decision-Making Objective (A)	Decision-Making Factors (B)	Decision-Making Indicators (C)
Optimal fertilization strategy plan for replacing nitrogen fertilizer with biogas slurry (A)	Soil nutrients (B1)	TN (C1)
		TN (C2)
		AP (C3)
		AK (C3)
		SOM (C4)
		AN (C5)
		PH (C6)
	Soil enzyme activity (B2)	CAT (C7)
		UE (C8)
		SC (C9)
		NP (C10)
	Soil bacterial diversity (B3)	B_sobs (C11)
		B_shannon (C12)
		B_simpson (C13)
	Soil fungal diversity (B4)	F_sobs (C14)
		F_shannon (C15)
		F_simpson (C16)
	Wheat yield and quality (B5)	Effective Spikes (C17)
		Thousand-Grain Weight (C18)
		Amylose (C19)
		Crude Protein (C20)

B_sobs: bacteria observed species; B_shannon: bacteria Shannon index; B_simpson: bacteria Simpson index; F_sobs: fungi observed species; F_shannon: fungi Shannon index; F_simpson: fungi Simpson index.

**Table 5:** Saaty 1–9 ratio-scale and method-scale values.

Relative Importance	Definition
1	Equally important
3	Slightly more important
5	Significantly more important
7	Clearly more important
9	Absolutely more important
2, 4, 6, 8	Intermediate value between two adjacent judgments
1/3	Slightly less important
1/5	Significantly less important
1/7	Absolutely less important
1/9	Absolutely less important
1/2, 1/4, 1/6, 1/8	Intermediate value between two adjacent judgments

**Table 6:** Effect of different fertilization programs on yield indicators of wheat.

Treat	Number of Effective Spikes m^−2^	Thousand-Grain Weight g	Amylose mg·g^−1^	Crude Protein g·kg^−1^	Yield kg·ha^−1^
CK	452.00 ± 43.27 a	47.20 ± 0.46 a	615.12 ± 18.02 b	131.39 ± 1.14 b	8970.67 ± 896.19 a
CF	505.33 ± 34.02 a	50.38 ± 0.82 a	514.73 ± 3.33 c	133.59 ± 1.74 b	9632.57 ± 720.53 a
S1	481.33 ± 85.54 a	49.07 ± 3.50 a	521.93 ± 3.34 c	123.12 ± 2.25 c	9120 ± 617.19 a
S2	468.00 ± 13.86 a	49.28 ± 3.66 a	528.91 ± 2.76 c	138.58 ± 2.01 a	8768 ± 532.59 a
S3	498.67 ± 29.48 a	50.65 ± 1.83 a	635.13 ± 14.24 a	124.31 ± 4.06 c	9461.33 ± 352.48 a

The same letters in the same column indicate that the differences between different treatments are not significant, while different letters indicate significant differences.

**Table 7:** A—B Consistency check and local weight values.

A—B	Soil Nutrition	Soil Enzyme Activities	Bacterial Diversity	Fungal Diversity	Wheat Quality	Local Weight Values
Soil nutrition	1	2	2	2	1	0.2857
Soil enzyme activities	1/2	1	1	1	1/2	0.1429
Bacterial diversity	1/2	1	1	1	1/2	0.1429
Fungal diversity	1/2	1	1	1	1/2	0.1429
Wheat quality	1	2	2	2	1	0.2857

Note: λ_max_ = 5, CI = 0, RI = 1.120, CR = 0 < 0.1.

**Table 8:** B1—C Consistency check and local weight values.

B1—C	TN	AP	AK	SOM	AN	PH	Local Weight Values
TN	1	2	3	1	1	4	0.2423
AP	1/2	1	2	1/2	1/2	3	0.1369
AK	1/3	1/2	1	1/3	1/3	2	0.0829
SOM	1	2	3	1	1	4	0.2423
AN	1	2	3	1	1	4	0.2423
PH	1/4	1/3	1/2	1/4	1/4	1	0.0534

Note: λ_max_ = 6, CI = 0.0074, RI = 1.2400, CR = 0.0059 < 0.1.

**Table 9:** B2—C Consistency check and local weight values.

B2—C	CAT	UE	SC	NP	Local Weight Values
CAT	1	1/3	1	1/2	0.1409
UE	3	1	3	2	0.4554
SC	1	1/3	1	1/2	0.1409
NP	2	1/2	2	1	0.2628

Note: λ_max_ = 4, CI = 0.0035, RI = 0.9000, CR = 0.0038 < 0.1.

**Table 10:** B3—C Consistency check and local weight values.

B3—C	B_sobs	B_shannon	B_simpson	Local Weight Values
B_sobs	1	2	2	0.5
B_shannon	1/2	1	1	0.25
B_simpson	1/2	1	1	0.25

Note: λ_max_ = 3, CI = 0, RI = 0.5800, CR = 0 < 0.1.

**Table 11:** B4—C Consistency check and local weight values.

B4—C	F_sobs	F_shannon	F_simpson	Local Weight Values
F_sobs	1	2	2	0.50
F_shannon	1/2	1	1	0.25
F_simpson	1/2	1	1	0.25

Note: λ_max_ = 3, CI = 0, RI = 0.5800, CR = 0 < 0.1.

**Table 12:** B5—C Consistency check and local weight values.

B5—C	Effective Spikes	Thousand-Grain Weight	Amylose	Crude Protein	Local Weight Values
Effective Spikes	1	1	2	2	0.3333
Thousand-Grain Weight	1	1	2	2	0.3333
Amylose	1/2	1/2	1	1	0.1667
Crude Protein	1/2	1/2	1	1	0.1667

Note: λ_max_ = 4, CI = 0, RI = 0.9000, CR = 0 < 0.1.

## Data Availability

The original contributions presented in this study are included in this article. Further inquiries can be directed to the corresponding author.
